# Development of Functional Abilities Assessment in Paediatric Oncology (FAAP-O) Scale for Children and Adolescents Affected by Cancer

**DOI:** 10.3390/children12091163

**Published:** 2025-09-01

**Authors:** Francesca Rossi, Monica Valle, Giulia Carlucci, Marco Tofani, Giovanni Galeoto, Paola Berchialla, Veronica Sciannameo, Marco Clari, Mario Cardano, Federica Nota, Daniele Bertin, Annalisa Calcagno, Roberto Casalaz, Margherita Cerboneschi, Marta Cervo, Annalisa Cornelli, Morena Delle Fave, Maria Esposito, Miriana Ferrarese, Paola Imazio, Maria Lorenzon, Lucia Longo, Gabriella Naretto, Nicoletta Orsini, Daniele Panzeri, Chiara Pellegrini, Michela Peranzoni, Fabiola Picone, Marco Rabusin, Antonio Trabacca, Claudia Zigrino, Andrea Martinuzzi, Franca Fagioli, Federica Ricci

**Affiliations:** 1 Rehabilitation Service, Public Health and Paediatric Sciences Department, A.O.U. Città della Salute e della Scienza—Regina Margherita Children Hospital, 10126 Turin, Italy; 2 Department of Public Health and Paediatric Sciences, University of Turin, 10124 Turin, Italy; monica.valle@unito.it (M.V.); marco.clari@unito.it (M.C.); m.espo79@libero.it (M.E.); andrea.martinuzzi@unito.it (A.M.); federica.ricci@unito.it (F.R.); 3 Health Professions of Rehabilitation Sciences Master’s Degree Course, Clinical and Biological Sciences Department, University of Turin, 10043 Orbassano, Italy; gcarlucci@asl.at.it (G.C.); miriana.ferrarese@maggioreosp.novara.it (M.F.); riabilitazione.motoria@ugi-torino.it (L.L.); 4 Department of Life Sciences, Health and Healthcare Professions, Università degli Studi “Link Campus University”, via del Casale di San Pio V 44, 00165 Rome, Italy; m.tofani@unilink.it; 5 Management and Diagnostic Innovations & Clinical Pathways Research Area, Neurorehabilitation and Adapted Physical Activity Day Hospital, IRCCS, Bambino Gesù Children’s Hospital, 00165 Rome, Italy; 6 Department of Human Neurosciences, Sapienza University of Rome, 00185 Rome, Italy; giovanni.galeoto@uniroma1.it; 7 IRCSS Neuromed, 86077 Pozzilli, Italy; 8 Centre of Biostatistics, Epidemiology and Public Health, Department of Clinical and Biological Sciences, University of Turin, 10124 Turin, Italy; paola.berchialla@unito.it (P.B.); veronica.sciannameo@gmail.com (V.S.); 9 Department of Cultures, Politics, and Society, University of Turin, 10124 Turin, Italy; mario.cardano@unito.it (M.C.); federica.nota@unito.it (F.N.); 10 Paediatric Oncohematology, Stem Cell Transplantation and Cell Therapy Division, A.O.U. Città della Salute e della Scienza—Regina Margherita Children’s Hospital, 10126 Turin, Italy; daniele.bertin@unito.it (D.B.); franca.fagioli@unito.it (F.F.); 11 Physical Therapy and Rehabilitation Department, Children’s Hospital Giannina Gaslini, 16147 Genoa, Italy; annalisacalcagno@gaslini.org (A.C.); nicoletta_orsini@yahoo.it (N.O.); 12 Paediatric Oncohematology Unit, Institute for Maternal and Child Health—IRCCS Burlo Garofolo, 34137 Trieste, Italy; roberto.casalaz@burlo.trieste.it (R.C.); marco.rabusin@burlo.trieste.it (M.R.); 13 Rehabilitation Department, IRCCS, Meyer Children’s Hospital, 50139 Firenze, Italy; m.cerboneschi@meyer.it (M.C.); marta.cervo@meyer.it (M.C.); fabiola.picone@meyer.it (F.P.); 14 Pediatric Oncology Department, ASST Papa Giovanni XXIII, 24127 Bergamo, Italy; congiulia@mailcertificata.org; 15 Neuro-Oncological Rehabilitation Unit, Scientific Institute IRCCS E. Medea, 23842 Bosisio Parini, Italy; morena.dellefave@lanostrafamiglia.it (M.D.F.); daniele.panzeri@lanostrafamiglia.it (D.P.); 16 Rehabilitation Department of Paediatric Orthopedics Unit, A.O.U. Città della Salute e della Scienza—Regina Margherita Children’s Hospital, 10126 Turin, Italy; paola.imazio@unito.it (P.I.); gabriellaelena.naretto@unito.it (G.N.); 17 Neuromotor Rehabilitation Unit, Scientific Institute, IRCCS E. Medea, 31015 Conegliano, Italy; maria.lorenzon@lanostrafamiglia.it; 18 Palliative Care, Pain Therapy and Rehabilitation Unit, Fondazione IRCCS Istituto Nazionale dei Tumori, 20133 Milan, Italy; chiara.pellegrini@asl.novara.it; 19 Department of Physiotherapy, Hospital of Bolzano, 39100 Bolzano, Italy; michela.peranzoni@sabes.it; 20 Scientific Institute IRCCS. “E. Medea”, Scientific Direction, 23824 Bosisio Parini, Italy; antonio.trabacca@lanostrafamiglia.it; 21 Unit for Severe Disabilities in Developmental Age and Young Adults Associazione “La Nostra Famiglia”, IRCCS “E. Medea”, Scientific Hospital for Neurorehabilitation, 72100 Brindisi, Italy; claudia.zigrino@lanostrafamiglia.it

**Keywords:** physical therapy, rehabilitation evaluation, GMFM-88, FAAP-O, paediatric oncology

## Abstract

**Highlights:**

**What are the main findings?**

- Starting from the Gross Motor Function Measure Scale (GMFM-88), the Functional Abilities Assessment in Paediatric Oncology (FAAP-O) has been developed through a multi-phase mixed-methods multicentre study.
- The FAAP-O psychometric properties which have been investigated are strong.

**What is the implications of the main findings?**

- A new scale specifically validated to assess functional abilities in children/adolescents (6 months–18 years old) with cancer is provided.
- FAAP-O is an objective rehabilitation assessment tool, which can support both clinical practice and research.

**Abstract:**

**Background/Objectives**: Functional abilities are fundamental for social participation. However, functional abilities assessment tools validated for children and adolescents undergoing cancer treatment are lacking. A preliminary validation of the Gross Motor Function Measure-88 (GMFM-88) scale for children with cancer found that some items are unable to discriminate or distinguish between different gross motor function levels. This study aims to develop and validate the Functional Abilities Assessment in Paediatric Oncology (FAAP-O), starting with GMFM-88. **Methods**: This multicentre study (ClinicalTrials.gov Identifier: NCT04862130) involved children and adolescents (6 months–18 years old) diagnosed with cancer, in any phase of treatment or less than 1 year off therapy. A multiphase mixed-methods design was employed. The content validity of each item of GMFM-88 for the paediatric oncology population was calculated with the Content Validity Ratio (CVR). Items with a CVR score ≥ 0.70 were included in the FAAP-O scale. Other items with a score between 0 and 0.69 were selected for their relevance by consensus with five focus groups and a plenary meeting. The FAAP-O items set was organised in five dimensions by exploratory factor analyses. The calculation of FAAP-O internal consistency was made using Cronbach’s α while inter/intra-rater reliability was assessed by intraclass correlation coefficients (ICCs). **Results**: The study involved 217 subjects. The FAAP-O included 36 items; its internal consistency was good in each dimension (0.90 ≤ α ≤ 0.96) and its inter/intra-rater reliability was excellent (ICC > 0.90). **Conclusions**: A new specifically validated scale to assess functional abilities in children and adolescents with cancer is provided. Validated tools are necessary for specific, objective rehabilitation assessments, which are fundamental in clinical practice and research.

## 1. Introduction

During the past five decades, remarkable progress has been made in the development of treatments for childhood cancer paediatric malignancies [1]. More than 80% of children with cancer who have access to contemporary therapies are expected to survive into adulthood [2]. According to the growing population of survivors, in recent decades, paediatric cancer rehabilitation practice and research have been recognised as supportive care for children experiencing the adverse physical effects of aggressive and prolonged cancer treatment [3]. Many children present motor impairments both at diagnosis [4,5], and during treatment [6], when different sequelae may occur and interfere with normal physical functioning [7,8]. Indeed, the therapies responsible for increased survival rates can also produce adverse, long-term, health-related outcomes, referred to as late effects, which may appear months to years after the completion of cancer treatment [1].

Physical therapy interventions in children and adolescents affected by cancer are focused on symptom relief and the compensation of therapy-related side effects [9]. Rehabilitation interventions for children with cancer aim to restore their functional abilities and mobility [9], to improve independence and the ability to participate in age-appropriate activities. According to the International Classification of Functioning, Disability and Health—version for Children and Youth (ICF-CY) [10], important outcomes therefore include improvements stemming from changes in patient impairment, activity limitations, and restricted social participation, which is a consequence impact their quality of life [11]. The ICF supports the move from traditional physiological and impairment-based measurement tools (e.g., range of motion and muscle strength) towards a more holistic view of health and a greater emphasis on measuring levels of function, activity, and participation [11].

Functional abilities are fundamental for the social participation of children, adolescents, and their families. However, there is a lack of assessment tools specifically created to evaluate gross motor function in children with different cancer diagnoses and in different phases of treatment. Rehabilitation evaluation is fundamental, for both research and clinical practice. A clearly documented record of treatment outcomes, using standardised outcome measures, provides the rehabilitation team with meaningful data [11] to assess the intervention effectiveness. On the other hand, the information derived from outcome measures supports clinical decisions regarding treatment options [11]. Since the psychometric properties of a measurement tool are closely linked to the specific population in which it is applied [11], it is would be necessary to use the assessment tools that have been validated for the specific population of children with cancer.

The internal consistency and floor/ceiling effects of the Gross Motor Function Measure-88 (GMFM-88) for use with children affected by cancer have recently been evaluated. However, the preliminary validation study found that some items are unable to discriminate between different gross motor function levels [12]. These findings underline how critical it is to have an assessment tool created and validated to evaluate specific gross motor function impairments related to cancer treatment. Rehabilitation evaluation in children and adolescents affected by cancer is challenging, due both to their clinical condition (e.g., sickness, fatigue, irritability, pain) and to the evaluation setting (e.g., day hospital, isolation regimen). These conditions may preclude the feasibility of the rehabilitation evaluation, both in terms of individual tolerability as well as the use of assessment equipment which is not always accessible. The Gross Motor Function Measure, a scale measure also known as the GMFM-88, is a criterion-referenced observational tool measure specifically developed to evaluate the changes in gross motor function over time in children with cerebral palsy [13]. Since the widely known tool GMFM-88 is used by many therapists, they already have the GMFM manual necessary for its use (the GMFM-88 manual is available at: https://www.mackeith.co.uk/ (accessed on 23 August 2025)); also, as its use requires equipment commonly used in a rehabilitation service [12], it can be used for multicentre studies. However, the GMFM-88 preliminary validation for children affected by cancer was performed to evaluate the internal consistency and floor/ceiling effects, finding that some items are unable to discriminate between different gross motor function levels, beyond the validation results. Two further issues emerged from the experts’ discussion. One was the long duration of GMFM-88 administration—the GMFM-88 includes 88 items divided into five subdimensions—which can be difficult to perform in some phases of cancer treatment or for subjects affected by fatigue. The other issue is that the GMFM-88 scoring method does not consent for the weighting of “not tested” items as anything other than 0. This could lead to an underestimation of the overall score and, therefore, a distortion of the score.

The goal of this multicentre study was to develop and validate an appropriate gross motor function assessment tool for children and adolescents (age range: 6 months–18 years) affected by different kinds of cancer and undergoing various treatment phases, starting with GMFM-88: the Functional Abilities Assessment in Paediatric Oncology (FAAP-O).

Concerning the objective, the research group hypothesised that (1) the FAAP-O would have a robust internal consistency for the target population of children with cancer; (2) all the FAAP-O items would positively contribute to determining the total score; and (3) the FAAP-O could discriminate between patients with different cancer diagnoses and treatment phases.

## 2. Materials and Methods

This article was prepared according to the COSMIN checklist [14].

### 2.1. Study Group

This observational multicentre study was conducted by a research group including neuro- and psychomotor therapists of developmental age (TNPEEs), physiotherapists, child neuropsychiatrists, physiatrists, oncologists, and biostatisticians from the Rehabilitation Working Group of the Italian Association of Paediatric Hematology and Oncology (AIEOP) [15]. Collaboration was carried out with a nurse researcher, two sociologists, and the Rehabilitation and Outcome Measures Assessment (ROMA) Association.

The study involved ten Italian clinical centres, with seven belonging to the AIEOP network and three external centres taking care of these children/adolescents with cancer in the community. The process involved an expert panel (EP) including 14 therapists of the research group, 3 of whom were TNPEEs and 11 of which were physiotherapists. The EP members had different expertise in the rehabilitation of paediatric patients affected by cancer, in terms of cancer type, phase of treatment, and rehabilitation setting. All of the EP members are experienced with different age ranges. The evaluation panel underwent a six-hour GMFM-88 training session to ensure alignment and reliability across raters.

The study project was approved by the Ethical Committee of the coordinator centre (Comitato Etico Interaziendale AOU Città della Salute e della Scienza di Torino, AO Ordine Mauriziano e ASL Città di Torino) and by all other Ethical Committees of the centres participating in the study. The study was conducted according to the Declaration of Helsinki [16] and was registered on Clinicaltrials.gov (NCT04862130).

This study is part of a larger study designed to evaluate gross motor function in children and adolescents affected by cancer. Data from the first phase of the study were published previously [12].

### 2.2. Sampling

Sample size was determined by analysing previous validation studies and according to the statistical analysis intended to be performed [12], as reported in the previous publication [12]. The minimum sample size resulted was 197 subjects.

The sample recruitment has already been described in the paper reporting the first part of the study [12]. The convenience sample met the following inclusion criteria: (1) to be children or adolescents (age range 6 months–18 years), (2) to have a confirmed diagnosis of cancer, and (3) to have been on treatment for less than 1 year off therapy. Exclusion criteria regarded the inability to collaborate with simple requests and not having signed the informed written consent.

### 2.3. Study Design

The observational multicentre study followed a multiphase mixed-methods design. It consisted of five main phases. Phase I explored the content validity of each item of the GMFM-88 for the paediatric oncology population. Phase II identified the items that could better discriminate between different levels of gross motor function, and which could obtain the maximum internal consistency for the target population. Phase III validated the final item-set of the FAAP-O scale and defined the method of scoring. Phase IV performed the statistical confirmation of the extracted items of the FAAP-O. Phase V included psychometric testing to establish the internal consistency, the inter- and intra-reliability, and the preliminary discriminative ability/cross-cultural validity of the FAAP-O scale.

### 2.4. Procedures and Data Collection

Recruitment strategies are reported elsewhere [12]. Data regarding participants’ socio-demographic and oncological characteristics (age at evaluation, sex, type of cancer, treatment phase) were collected in a paper case report form and then inserted in an Excel dataset. Socio-demographic information was analysed with descriptive statistics, using frequency, mean (SD), and median (IQR) when appropriate.

Phase I: GMFM-88 content validity assessment.

The objective of Phase I was to identify which items of GMFM-88 reach a content validity score that indicates agreement among EP members, thus allowing inclusion in the scale. Content validity was assessed using the content validity ratio (CVR). The EP members evaluated each item of the GMFM-88 following a 4-point scale: 4 = highly relevant; 3 = quite relevant or highly relevant but needs rewording; 2 = somewhat relevant; and 1 = not relevant. The CVR can range between −1 and +1, and a value of 0 means that half the panel feel that the item is essential [17]. To ensure that the results’ robustness were not due to chance, a value of 0.70 was established for fourteen raters. Items with CVR values lower than 0 were discarded and those with CVR values ≥ 0.70 were included. Items whose CVR values were between 0.00 and 0.69 were discussed in Phase II.

Phase II: Content validity refinement.

The objective of Phase II was to identify which items had achieved CVR values between 0.00 and 0.69 in Phase I, which could better discriminate between different levels of gross motor function in the paediatric oncology population.

To carry out the qualitative analysis of experts’ opinions, five online focus groups were conducted, one for each GMFM-88 dimension. Each focus group hosted five EP members [18] with expertise in rehabilitation intervention in different types of cancer and settings during the disease trajectory and off-therapy. The focus groups were conducted by a nurse researcher supported by two external TNPEE observers.

During the focus groups, the EP participants were asked to examine the items and to evaluate their significance and relevance in the evaluation of paediatric oncology subjects affected by cancer, in their applicability in clinical practice, and in their ability to evaluate and objectify change. Consensus for item inclusion or exclusion was considered acceptable when the whole panel was unanimous. If complete agreement was not reached, the decision was postponed to the final online plenary meeting.

The focus groups were videotaped and transcribed verbatim.

Phase III: Final FAAP-O item set and method of scoring definition.

The objective of Phase III was to validate the final item set of the FAAP-O scale. A plenary meeting was performed, in which therapists could discuss the necessity for the final inclusion or exclusion of the items that did not reach consensus in the focus groups. Each item was to be approved unanimously while all experts were requested to outline the irrelevance and redundancy of each individual item which suggested its elimination from the scale. The final decision on the approval of GMFM-88 items that were included and excluded after the focus groups was evaluated through a survey. The participants were to respond “yes” or “no” to the question “Do you wish to include/exclude this item in the FAAP-O Scale?”. It was decided to accept inclusion of those items which reached consensus among ≥70% of responders.

Furthermore, during the plenary meeting, a discussion about how to manage non-tested items was performed.

The plenary meeting was videotaped and transcribed verbatim.

Phase IV: Statistical confirmation of the extracted items of the FAAP-O.

Exploratory factor analysis (EFA) was adopted to confirm the dimensional structure of the item-set selected for the FAAP-O. Since the theoretical framework was insufficiently specific to stipulate an a priori measurement model, the EFA provided an empirical route to uncover the smallest number of latent factors that adequately accounted for the observed inter-item correlations. Prior to extraction, the overall sampling adequacy was verified using the Kaiser–Meyer–Olkin (KMO) statistic: values ≥ 0.60 were deemed the minimum threshold [19]. Bartlett’s test of sphericity tested the null hypothesis that the correlation matrix was an identity matrix, and only when this test was significant (*p* < 0.05) did the matrix qualify for EFA [20]. Principal axis factoring, robust to the non-normal distributions typical of clinical data, was employed, and factors with eigenvalues greater than 1.00 were initially retained; this rule was triangulated with parallel analysis, scree-plot inspection, and substantive interpretability. Following extraction, a Promax oblique rotation was applied to obtain a simplified, interpretable structure consistent with the anticipated correlation among motor domains. Salient loadings were defined as 0.40 or higher, with cross-loading differences of at least 0.20 required for an item to be assigned unambiguously to a single factor; items with low communalities (<0.30) or problematic cross-loadings were iteratively reconsidered or discarded [21]. Finally, statistical decisions were weighted against clinical coherence: factor plausibility was debated in a second expert round, ensuring that the finalised FAAP-O embodied both the empirically derived structure and the pragmatic realities of paediatric oncology rehabilitation.

Phase V: Psychometric properties of FAAP-O.

The evaluation of the internal consistency, the inter- and intra-rater reliability, and the preliminary discriminative ability/cross-cultural validity was carried out to assess the preliminary psychometric properties of the FAAP-O scale.

Specifically, internal consistency was examined using Cronbach’s alpha coefficient, which assesses the extent to which the items within the FAAP-O consistently measure the intended construct—namely functional motor abilities. A Cronbach’s alpha value of 0.70 or higher was considered indicative of acceptable internal consistency [22].

Reliability was calculated by scoring 9 videotaped FAAP-O evaluations, including 3 subjects affected by leukaemia, 3 by central nervous system (CNS) tumours, and 3 by bone cancer, all of different ages and in various phases of treatment. The assessment was made using the Intraclass Correlation Coefficient (ICC) of both inter-rater reliability, which quantifies the degree to which independent clinicians assign concordant scores when assessing the same individual, and intra-rater reliability, which reflects the consistency of the scores assigned by the same clinician across repeated assessments. Intra-rater reliability was calculated by repeating twice the scoring of the same video, with a timeframe of two weeks between the first and the second rating. An ICC value of ≥0.70 was considered acceptable, with higher values reflecting stronger agreement between raters [23].

Discriminative ability was investigated to determine whether FAAP-O could distinguish between subgroups of the paediatric oncology population based on clinical characteristics. Comparisons were made across different phases of the cancer trajectory. A valid discriminative instrument should yield distinct score distributions among clinically relevant subgroups, thereby reflecting the functional differences attributable to treatment intensity or disease stage [24]. Cross-cultural validity, in this context, was represented by the ability of FAAP-O to measure the functional motor abilities consistently across various diagnostic groups within paediatric oncology. Cross-cultural validity ensures that the scale captures the same underlying construct regardless of the specific type of neoplastic condition, allowing for meaningful comparisons and the external validity and generalisability of the results across heterogeneous clinical populations [25]. Both discriminative ability and cross-cultural validity were then compared with the original version of GMFM-88.

Since the total FAAP-O score did not follow a normal distribution, a Kruskal–Wallis H test was conducted to assess the differences across subgroups. After observing a statistically significant omnibus effect, post hoc pairwise comparisons were performed using the Dunn Bonferroni approach, as implemented in SPSS (version 27, IBM Corp., Armonk, NY, USA). This method is equivalent to conducting Mann–Whitney U tests between all possible group pairs, with Bonferroni-adjusted *p*-values to control for a type I error due to multiple testing. Adjusted significance levels below *p* < 0.05 were considered indicative of statistically significant differences between groups.

## 3. Results

The GMFM-88 was administered to 217 children with a median age of 6.9 years [IQR 2.7–11.3]. Males represented 53.3% of the sample, the diagnosis of CNS tumours was reported in 55.8% of the subjects, and the most represented treatment phase was ‘on treatment’ (66.8%). Sample characteristics are summarised in Table 1.

### 3.1. Phase I: GMFM-88 Content Validity Assessment

Ten items (11.3%) of the eighty-eight potential items of GMFM-88 scale reached the threshold of validation (CVR ≥ 0.70). Twenty-four items (27.3%) did not reach the minimum threshold (CVR < 0), and fifty-four (61.4%) did not reach consensus (0 ≤ CVR < 0.70), despite obtaining acceptable agreement and remaining potential core items to be reconsidered in Phase II (Table 2). Item definition is reported in Table S1 in the Supplementary Materials.

- N_e_ = number of raters who consider the item essential (rating of 3 or 4);
- green cell = CVR > 0.70;
- red cell = CVR < 0.

### 3.2. Phase II: Content Validity Refinement

Fifty-four items with a CVR score between 0 and 0.70 (identified in phase I) were discussed in five focus groups, one for each dimension of GMFM-88. The five focus groups were homogeneous in terms of the participants’ expertise. Two items already included (24—*Sitting on mat, maintains, arms free, 3 s* -, 56—*Standing: maintains, arms free, 20 s* -) and two already excluded (44—*Four point: crawls or hitches forward 1.8 m (6ft))* - and 81—*Standing: jumps forward 30 cm, both feet simultaneously* -) were reconsidered due to their similarity to other questionable items. Therefore, a total of 58 items were discussed. The discussions lasted about five hours.

During the focus groups, the discussion was mostly unanimous regarding the acceptance and exclusion of items in Dimension A. In the event of indecision, the above criteria were recalled during the session to reach an agreement.

The decisions concerning Dimension B were also almost unanimous; the experts had contrasting opinions only on items number 28 (*Right side sitting: maintains, arms free, 5 s*), 29 (*Left side sitting: maintains, arms free, 5 s*), and 36 (*On the floor: attains sitting on a small bench*). The discussion highlighted how patients affected by bone tumours could not perform tasks outlined in items 28 and 29 for a long time. Therefore, experts voted for their exclusion since the aim was to create a scale for the entire paediatric oncology population. Item 36 was presented alongside 35 (*Standing: attains sitting on a small bench*), 37 (*On the floor: attains sitting on large bench*), and 52 (*On the floor: pulls to standing at large bench*), which belonged to Dimension D. Debate arose over the fact that each of these items could be considered the evolution of the other, since they mostly evaluate the organisation of movement from sitting to standing or vice versa. Again, the indecisiveness depended on their applicability to the whole oncology population: choosing a more advanced item that showed more information meant compromising the assessment of children and adolescents with bone tumours. In the end, the panel opted for the inclusion of items number 35 and 36, which allow for the assessment of a larger number of patients since they are more accessible items.

Dimension C was the most debated; the panellists encountered some difficulties in setting aside their own specific clinical practice and considering the paediatric oncology population as a whole. In particular, the most discussed items were number 42 (*Four points: reaches forward with right arm, hand above shoulder level*), 43 (*Four points: reaches forward with left arm, hand above shoulder level*), 44, and 45 (*Four points: crawls reciprocally forward 1.8 m (6ft)*). Some experts advocated for the exclusion of items 42 and 43 in favour of 44 or 45, as dynamic balance inherently assumes static balance. Instead, other experts defended the inclusion of all three, because they evaluate different tasks and skills. Moreover, this group of experts believed that items 42 and 43 were easier than 44 and 45 to implement in clinical practice due to the space needed to perform the latter and their inappropriacy for adolescents. Some experts suggested retaining items 44 or 45, as they are significant for preschoolers. Ultimately, the panel failed to reach an agreement and deferred the decision to the plenary meeting.

The discussion for Dimension D was concise and nearly always resulted in unanimous decisions. The panel showed some indecision with respect to items 54 (*Standing: holding on to large bench with one hand, lifts right foot, 3 s*), 55 (*Standing: holding on to large bench with one hand, lifts left foot, 3 s*), and 56 (*Standing: maintains, arms free, 20 s*). Unable to merge different items, the team decided to keep items 57 (*Standing: lifts left foot, arms free, 10 s*) and 58 (*Standing: lifts right foot, arms free, 10 s*), which could be considered as an evolution of the ability to stand on one leg using a support. In this case, the decision to exclude items 54, 55, and 56 for this population was based on the risk of obtaining a score ceiling effect.

The last focus group dedicated to dimension E resulted in significant debate, especially for items 77 (*Standing: runs 4.5 m (15 ft), stops & returns*) and 80 (*Standing: jumps 30 cm (12 ft) high, both feet simultaneously*). Since the first item consists in running, some experts argued that it is hard to evaluate this ability in acute clinical settings. In the end, the panel opted for the inclusion of item 77 because it could assess changes in the patient over time. With reference to item 80, the experts found it challenging to evaluate it, due to practical constraints such as the complexity of the assessment in patients undergoing chemotherapy with reduced strength. The final choice was postponed to the plenary meeting, as removing this item from the scale would have meant eliminating the possibility to rate the ability to jump.

In phase II, out of 58 discussed items, 28 were excluded and 24 included, while the panel could not reach an agreement for 6 items: 4 of Dimension C (42–45) and 2 of Dimension E (80 and 81). The final decision for the latter was postponed to the plenary meeting in Phase III.

### 3.3. Phase III: Final FAAP-O Item Set and Method of Scoring Definition

The first four unresolved items (42–45) were discussed together since they have a common starting position (four points). All fourteen participants were in favour of excluding item 44 and retaining the other three. Items 80 and 81 were discussed together because both evaluated the ability to jump. Experts decided to exclude item 80 and retain item 81. At the conclusion of the plenary meeting, a total of 36 items were definitively approved for the FAAP-O scale.

Unanimous approval was obtained through a survey to confirm all the items selected for inclusion/exclusion (Table S1 in Supplementary Materials).

Regarding the scoring method, PE confirmed the need to weigh the untested items with a score different from 0. The scoring system was redefined by maintaining the original consistent generic 4-point ordinal scale to assess each item, rewording GMFM-88 score 0, and adding the “not tested” category. Scoring categories are reported in Appendix A, while the scoring calculator is reported in the Supplementary Materials (S2). The research group decided to weigh untested items using a normalised scoring formula.

$$\left[\left(medium\;score\;obtained\;in\;the\;non\;-\;tested\;item’s\;dimension-1\right)\right]\times 50$$

### 3.4. Phase IV: Statistical Confirmation of the Extracted Items of the FAAP-O

The EFA confirmed that the item set selected for the FAAP-O was structured in five dimensions (Appendix A), which explained 77% of the total variance (Table S2 in Supplementary Materials).

For all FAAP-O dimensions (except Dimensions 3 and 4), the EFA results were approved by experts. The PE voted to move item 36 (*On the floor: pulls to standing on a small bench*) from Dimension 3 to 4 since its starting position on a mat, similarly to most items in Dimension 4 as per EFA values.

**Figure 1:**
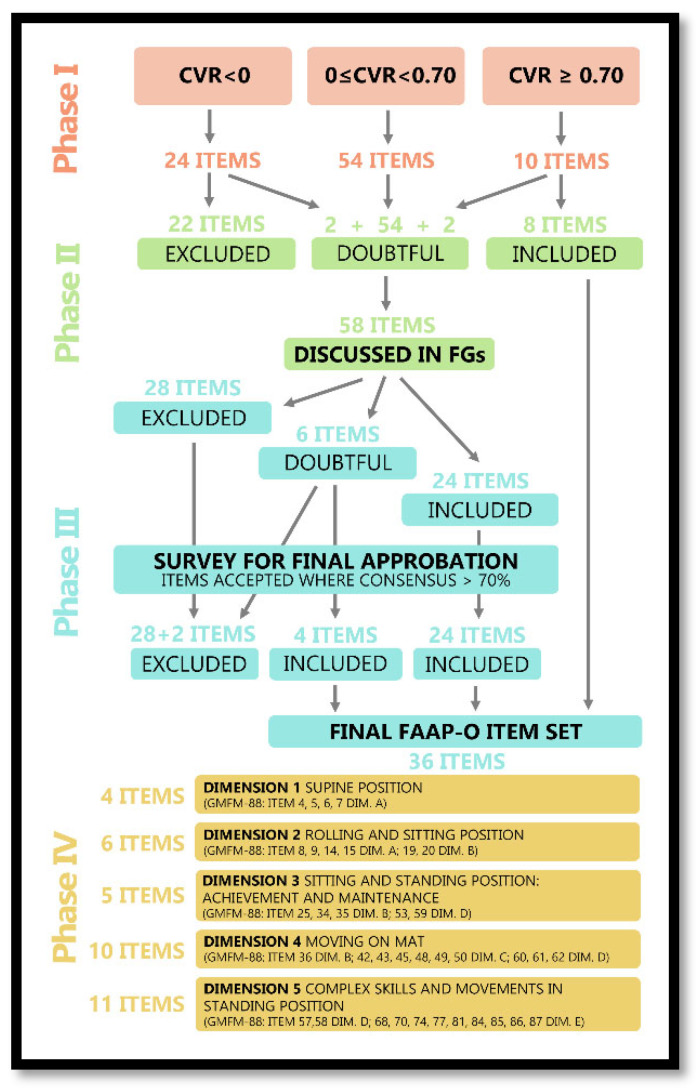
FAAP-O item set definition process.

The entire FAAP-O item set definition process is reported in Figure 1 and the final FAAP-O item set is reported in Appendix A.

### 3.5. Phase V: Psychometric Properties of FAAP-O

Internal consistency estimates revealed a Cronbach’s α of 0.95 for the FAAP-O total score. FAAP-O Cronbach’s α was 0.91 for Dimension 1, 0.94 for Dimension 2, 0.90 for Dimension 3, 0.96 for Dimension 4, and 0.94 for Dimension 5. The mean (SD) score and item-total statistics for each FAAP-O item are reported in Table 3. Item definition is reported in Table S1 in the Supplementary Materials.

FAAP-O reliability analysis with an ICC 95% CI was 0.99 for both inter and intra-reliability. The findings of ICC for each dimension are reported in Table 4.

Preliminary discriminative ability/cross-cultural validity were evaluated to determine whether the FAAP-O was effective in measuring gross motor function across tumour subgroups and treatment stages. The Kruskal–Wallis H test revealed a significant difference in the total FAAP-O scores across tumour subgroups (χ^2^(3) = 11.835, *p* = 0.008). The post hoc Dunn–Bonferroni pairwise comparisons showed that the children in the Leukaemia/Lymphoma Group had significantly higher scores compared to the CNS Tumours Group (adjusted *p* = 0.019) and marginally higher scores than the Other Solid Tumours Group (adjusted *p* = 0.046). The Leukaemia/Lymphoma Group reported higher total scores. No other comparisons reached statistical significance. The Kruskal–Wallis H test also revealed a statistically significant difference across groups (χ^2^(2) = 11.37, *p* = 0.003) for different treatment phases. Post hoc pairwise comparisons with Bonferroni correction showed that patients in the Post-Surgery Group reported significantly lower scores than those off-therapy (adj *p* = 0.002), and that patients in treatment had lower scores than those off-therapy (adj *p* = 0.036). No statistically significant difference was found between the Post-Surgery and the On-Treatment groups (adj *p* = 0.259). The same analysis was performed using GMFM-88. The Kruskal–Wallis H test was used to examine whether the total scores of GMFM-88 differed significantly across four tumour subtypes, such as Leukaemia/Lymphoma, CNS tumours, bone cancer, and other solid tumours. The test revealed a statistically significant difference among groups (χ^2^(3) = 10.477, *p* = 0.015). Post hoc pairwise comparisons with Bonferroni correction showed a statistically significant difference between the Leukaemia/Lymphoma Group and the CNS Group (adjusted *p* = 0.027), with the former Group reporting higher total scores. Other comparisons did not reach statistical significance. The test indicated a statistically significant difference among groups (χ^2^(2) = 12.165, *p* = 0.002) at different stages of treatment. Post hoc pairwise comparisons with Bonferroni correction revealed that patients in the Post-Surgery Group scored significantly lower than those off-therapy (adjusted *p* = 0.002) and also significantly lower than those on treatment (adjusted *p* = 0.021). No significant difference was found between the on-treatment and off-therapy groups (adjusted *p* = 0.302). A comparison between the main findings of FAAP-O and of GMFM-88 is summarised in Table 5 and Table 6.

## 4. Discussion

The results from the development and preliminary validation process of the FAAP-O scale, an assessment tool that identifies gross motor function levels in subjects from the age of 6 months to 18 years old, affected by various types of cancer and in different treatment phases, are reported. Most assessment tools validated for this population considered outcomes other than different from gross motor function [26,27,28] and all but one [26] regarded specific cancer sub-populations [27,28,29]. An assessment tool validated for a broad paediatric cancer population may enhance rehabilitation research and improve clinical practice.

FAAP-O is suitable for multicentre studies designed to involve subjects with all types of cancer. A recent systematic review [30] identified only four disease-specific clinical practice guidelines for paediatric oncology rehabilitation, focused on ALL [31], CNS tumours [32], Hodgkin’s lymphoma [33], and bone cancer [34]. It suggests that researchers cannot give any indications for clinical practice due to the paucity of studies involving other populations. The MOON-scale was developed for children affected by all cancer types [26]; however, this tool is not specifically focused on gross motor function and the equipment required for its administration is not easily available (e.g., a special pin box, a special wooden bar, and a handheld dynamometer).

FAAP-O is also suitable for multicentre studies designed to involve preschoolers. It is well known that cancer and antineoplastic treatments have an impact on children’s development, impairing fine and gross motor skills [35], yet this age range remains under-investigated. Only the GMFM-ALL has been validated for subjects above 5 years of age [29]. This assessment scale was made up of items selected solely from GMFM-88 Dimensions D and E, which require the ability to stand independently to perform all items but one, thus making it inappropriate for children under than 10 months old [36].

FAAP-O is suitable for multicentre studies performed in different settings. Since no specific equipment is required, this scale is adapted for use in resource-limited settings, such as day-hospitals and low-income countries. Indeed, FAAP-O can be completed during a rehabilitation session, making it suitable also for subjects in a day-hospital regimen. Furthermore, the possibility of recording “not tested” items, allows the use of FAAP-O also with children with movement restrictions (e.g., subjects with external ventricular derivation, amputation, or connected to infusion lines), suffering from treatment side effects (e.g., nausea, vomiting, fatigue), or in specific treatment phases (e.g., post-surgery, palliative care), without underestimating the overall score.

The use of both quantitative and qualitative methods allowed us to obtain an assessment tool with a robust internal consistency. Some authors report that acceptable values of α should be between 0.70 and to 0.95 [37] since a high coefficient α might suggest that some items are redundant [38]. Therefore, FAAP-O, where Cronbach’s alpha values range from 0.90 to 0.96, seems to be more focused on the construct of interest than GMFM-88 validated for children with cancer, where Cronbach’s alpha values range from 0.95 to 0.99 [12]. Statistical analysis revealed that the FAAP-O items positively contribute to determining the total score.

Referring to inter- and intra-reliability, since both achieved an ICC 95% CI value greater than 0.90, FAAP-O demonstrated excellent agreement between raters and for the same rater over time, confirming its reproducibility and stability [39].

Discriminative ability/cross-cultural validity found that FAAP-O is effective in discriminating gross motor function across tumour subgroups and treatment phases. Our analysis revealed that the Leukaemia/Lymphoma Group reported higher total scores. This result is in line with an extended paediatric cancer survivor study reporting that the highest prevalence of functional limitations was found in patients with bone sarcoma and brain tumours [40]. Furthermore, the Leukaemia/Lymphoma Group had significantly higher FAAP-O scores compared to the CNS Tumours Group. This could be related to the unique sequelae that children affected by CNS tumours may experience [41]. Indeed, in this sub-population, gross motor function can be impacted not only by the common anti-neoplastic treatment effects, but also by neurological deficits and cognitive impairments related both to cancer itself and to neuro-surgical and neuro-oncological treatments. Subjects in the Post-Surgery Group reported significantly lower FAAP-O scores than those off-therapy. The impact of surgery on gross motor function is high for both subjects undergoing neurosurgery and musculoskeletal surgery. In the post-operative phase, body structure surgery impacts, pain, psychological aspects, and medical restrictions highly limit functional abilities. Many studies report a progressive improvement in gross motor function over time following surgery. Subjects undergoing lower extremities reconstruction for musculoskeletal tumours progressively achieve better functional levels during the first post-operative year [42]. Children admitted to inpatient rehabilitation following brain tumour resection had significant improvements from admission to 3-month follow-up after discharge [43]. These findings confirm that gross motor abilities tend to improve as more time elapses after surgery, thereby justifying lower scores in children during the post-surgical phase compared to those who are off-therapy.

Subjects on treatment had lower FAAP-O scores than those off-therapy. This finding correlates with the fact that antineoplastic treatment is frequently accompanied by adverse events such as nausea, serious infections, organ damage and decreased bone density, but also decreased muscle strength and physical fitness [44] contributing to reduced physical functioning. Adults who have undergone childhood cancer treatment display high levels of sedentary behaviour and can experience lifelong impairment [45]. However, during acute treatment, there are more factors that can impact gross motor functions, justifying our results. Both FAAP-O and GMFM-88 can discriminate between off-therapy and Post-Surgery Groups. However, FAAP-O is also able to discriminate between off-therapy and on-treatment groups, while GMFM-88 can discriminate Post-Surgery from the On-treatment Groups. However, since surgery is a treatment modality used in a smaller minor number of subjects, and according to the aim to develop an assessment tool suitable for the paediatric cancer population as a whole, we can therefore assert that FAAP-O has better discriminative capacity than GMFM-88.

### Study Limitations

Our study presents some limitations. Firstly, the sample was composed of a reduced number of infants and children/adolescents affected by bone tumours, with a prevalence of participants with CNS cancers and undergoing active treatment. Furthermore, we did not evaluate some relevant FAAP-O psychometric proprieties, such as responsiveness to change and ceiling/floor effects; even though the findings of this study suggest that the FAAP-O demonstrates good psychometric properties, further analyses are required to confirm its relevance and applicability in the target population. In particular, future studies should investigate responsiveness to change, measurement error, potential floor and ceiling effects, and perform a stratified analysis among specific cancer subtypes groups. A more refined evaluation of the FAAP-O’s psychometric properties would further strengthen its role as a reliable outcome measure in rehabilitation settings for children with cancer.

Another limitation relates to the wide age range of participants. Since motor development varies substantially across this developmental span, age may influence FAAP-O scoring and interpretation. Future research should therefore explore age-specific analyses to improve interpretability across different age groups.

Finally, it would be very useful to develop reference values for healthy children under 5 years of age, to establish and weigh the discrepancy with expected development.

## 5. Conclusions

The FAAP-O scale is an assessment tool specifically developed to evaluate gross motor function in the paediatric oncology population during treatment and up to one year off-therapy. Gross motor function is relevant for participation and independence, and represents one of the most important goals of rehabilitation therapy. The relevance of FAAP-O outcomes, the low equipment resources required for its administration, as well as the possibility to weigh untested items based on the global ability level shown by the child in the other items of the same dimension all allow the use of the tool in many different contexts. The results suggest that the FAAP-O has potential value for clinical use, though further studies are warranted to strengthen the evidence base and address remaining limitations.

Although the FAAP-O is designed to be low-resource and adaptable, aspects such as administration time, training requirements, and inter-rater calibration are critical for its feasibility in real-world settings. In this study, the average administration time was approximately 30 min, and evaluators used the GMFM-88 manual for item scoring. The evaluation panel underwent a six-hour GMFM-88 training session to ensure alignment and reliability across raters. Specific training in GMFM-88 administration can be found at https://canchild.ca (accessed on 23 August 2025).

This tool would contribute to the advancement of paediatric oncology rehabilitation research by promoting the development of multicentre studies aimed at describing and verifying the effectiveness of physical therapy in achieving functional meaningful goals for both patients and their families.

## Figures and Tables

**Table 1:** Demographic characteristics of the participants (total 217).

**Age**	Years (IQR *)
Age at evaluation	6.9 years [IQR 2.7–11.3]
**Sex**	**N (%)**
Female	101 (46.5)
Male	116 (53.5)
**Type of cancer**	**N (%)**
Central nervous system tumours	121 (55.8)
Bone cancers	12 (5.5)
Leukaemia/lymphoma	63 (29)
Other solid tumours	21 (9.7)
**Treatment phase**	**N (%)**
On treatment	145 (66.8)
Off therapy	31 (14.3)
Post-surgery	41 (18.9)

* IQR = interquartile range.

**Table 2:** CVR results.

ITEM.	Ne	CVR		ITEM	Ne	CVR		ITEM	Ne	CVR
**1**	1	−0.8571429		**31**	8	0.1428571		**60**	13	0.8571429
**2**	5	−0.2857143		**32**	8	0.1428571		**61**	13	0.8571429
**3**	6	−0.1428571		**33**	3	−0.5714286		**62**	12	0.7142857
**4**	8	0.1428571		**34**	11	0.5714286		**63**	8	0.1428571
**5**	8	0.1428571		**35**	10	0.4285714		**64**	11	0.5714286
**6**	7	0.0000000		**36**	10	0.4285714		**65**	4	−0.4285714
**7**	8	0.1428571		**37**	7	0.0000000		**66**	4	−0.4285714
**8**	10	0.4285714		**38**	3	−0.5714286		**67**	6	−0.1428571
**9**	9	0.2857143		**39**	10	0.4285714		**68**	8	0.1428571
**10**	5	−0.2857143		**40**	8	0.1428571		**69**	9	0.2857143
**11**	3	−0.5714286		**41**	5	−0.2857143		**70**	13	0.8571429
**12**	7	0.0000000		**42**	10	0.4285714		**71**	7	0.0000000
**13**	7	0.0000000		**43**	10	0.4285714		**72**	7	0.0000000
**14**	7	0.0000000		**44**	5	−0.2857143		**73**	9	0.2857143
**15**	6	−0.1428571		**45**	9	0.2857143		**74**	10	0.4285714
**16**	3	−0.5714286		**46**	1	−0.8571429		**75**	7	0.0000000
**17**	3	−0.5714286		**47**	1	−0.8571429		**76**	7	0.0000000
**18**	7	0.0000000		**48**	10	0.4285714		**77**	10	0.4285714
**19**	7	0.0000000		**49**	13	0.8571429		**78**	8	0.1428571
**20**	6	−0.1428571		**50**	12	0.7142857		**79**	8	0.1428571
**21**	1	−0.8571429		**51**	7	0.0000000		**80**	7	0.0000000
**22**	8	0.1428571		**52**	11	0.5714286		**81**	5	−0.2857143
**23**	9	0.2857143		**53**	8	0.1428571		**82**	4	−0.4285714
**24**	12	0.7142857		**54**	9	0.2857143		**83**	4	−0.4285714
**25**	8	0.1428571		**55**	9	0.2857143		**84**	12	0.7142857
**26**	5	−0.2857143		**56**	13	0.8571429		**85**	11	0.5714286
**27**	5	−0.2857143		**57**	10	0.4285714		**86**	11	0.5714286
**28**	7	0.0000000		**58**	10	0.4285714		**87**	10	0.4285714
**29**	7	0.0000000		**59**	14	1.0000000		**88**	2	−0.7142857
**30**	4	−0.4285714								

Legend: N_e_ = number of raters who consider the item essential (rating of 3 or 4); green cell = CVR > 0.70; red cell = CVR < 0.

**Table 3:** FAAP-O mean (SD) score and item-total statistics.

FAAP-O	Mean	Standard Deviation	Scale Mean if Item Deleted	Scale Variance if Item Deleted	Corrected Item-Total Correlation	Cronbach’s Alpha if Item Deleted
ITEM1	2.60	0.894	63.50	1075.507	0.491	0.976
ITEM2	2.62	0.876	63.48	1073.274	0.541	0.976
ITEM3	2.75	0.810	63.35	1083.372	0.396	0.976
ITEM4	2.68	0.913	63.42	1076.179	0.469	0.976
ITEM5	2.31	1.169	63.79	1049.694	0.714	0.975
ITEM6	2.28	1.173	63.81	1046.713	0.752	0.975
ITEM7	2.06	1.383	64.03	1035.181	0.765	0.975
ITEM8	2.07	1.365	64.02	1035.414	0.772	0.975
ITEM9	2.17	1.301	63.93	1043.343	0.715	0.975
ITEM10	2.21	1.280	63.88	1046.218	0.692	0.975
ITEM11	2.57	0.962	63.52	1066.809	0.595	0.976
ITEM 12	2.52	1.043	63.57	1060.301	0.644	0.975
ITEM 13	2.14	1.329	63.95	1035.105	0.798	0.975
ITEM 14	1.75	1.440	64.34	1030.820	0.781	0.975
ITEM 15	1.94	1.366	64.16	1033.863	0.790	0.975
ITEM 16	1.95	1.368	64.14	1032.328	0.807	0.975
ITEM 17	1.91	1.390	64.18	1044.260	0.656	0.975
ITEM 18	1.96	1.311	64.13	1035.667	0.803	0.975
ITEM 19	1.62	1.409	64.48	1026.241	0.852	0.975
ITEM 20	1.59	1.408	64.50	1026.242	0.853	0.975
ITEM 21	2.37	1.185	63.73	1047.986	0.726	0.975
ITEM 22	1.25	1.262	64.84	1045.668	0.709	0.975
ITEM 23	1.26	1.243	64.83	1045.482	0.723	0.975
ITEM 24	1.90	1.304	64.19	1032.902	0.841	0.975
ITEM 25	1.44	1.263	64.65	1037.977	0.805	0.975
ITEM 26	1.44	1.249	64.65	1039.670	0.794	0.975
ITEM 27	1.53	1.272	64.56	1038.666	0.791	0.975
ITEM 28	2.16	1.327	63.94	1037.345	0.772	0.975
ITEM 29	1.94	1.411	64.15	1030.381	0.803	0.975
ITEM 30	0.93	1.229	65.17	1050.995	0.660	0.975
ITEM 31	0.85	1.256	65.24	1052.267	0.629	0.976
ITEM 32	0.87	1.214	65.23	1050.892	0.670	0.975
ITEM 33	1.42	1.431	64.68	1033.727	0.753	0.975
ITEM 34	1.27	1.372	64.82	1034.586	0.778	0.975
ITEM 35	0.90	1.286	65.19	1047.422	0.673	0.975
ITEM 36	0.88	1.276	65.21	1048.463	0.666	0.975

**Table 4:** Inter- and intra-rater reliability of the FAAP-O.

Reliability	Inter-Rater		Intra-Rater	
	ICC	95% CI	ICC	95% CI
**Dimension 1** (4 item)	**1**	1–1	**0.996**	0.988–0.999
**Dimension 2** (6 items)	**0.993**	0.972–0.998	**0.937**	0.811–0.989
**Dimension 3** (5 Items)	**0.994**	0.977–0.999	**0.984**	0.952–0.995
**Dimension 4** (10 items)	**0.995**	0.981–0.999	**0.983**	0.984–0.994
**Dimension 5** (11 items)	**0.998**	0.993–1	**0.955**	0.865–0.985
**Total Score** (36 items)	**0.998**	0.993–1	**0.991**	0.974–0.997

Legend: ICC = intraclass correlation coefficients; CI = confidence interval.

**Table 5:** FAAP-O preliminary discriminative ability/cross-cultural validity for neoplastic forms (*p* values).

***GMFM-88***				
**Neoplastic forms**	***Other Solid*** ***Tumours***	***Leukaemia***	***Bone Tumours***	***Central Nervous*** ***System Tumours***
Other solid tumours	/	0.006 *	0.404	0.181
Leukaemia	0.006 *	/	0.836	0.120
Bone tumours	0.404	0.836	/	0.720
Central nervous system tumours	0.181	0.120	0.720	/
***FAAP-O***				
**Neoplastic forms**	***Other solid tumours***	***Leukaemia***	***Bone tumours***	***Central nervous system tumours***
Other solid tumours	/	0.03 *	0.309	0.186
Leukaemia	0.03 *	/	0.956	0.004 *
Bone tumours	0.309	0.956	/	0.730
Central nervous system tumours	0.186	0.004 *	0.730	/

Legend: * *p* < 0.05.

**Table 6:** FAAP-O preliminary discriminative ability/cross-cultural validity for treatment phases (*p* values).

***GMFM-88***			
**Phases**	***On Treatment***	***Off-Therapy***	***Post-Surgery***
On treatment	**/**	0.009 **	0.02 *
Off-therapy	0.009 **	**/**	0.001 **
Post-surgery	0.02 *	0.001 **	**/**
***FAAP-O***			
**Phases**	***On treatment***	***Off-therapy***	***Post-surgery***
On treatment	**/**	0.01 *	0.01 *
Off-therapy	0.01 *	**/**	0.002 **
Post-surgery	0.01 *	0.002 **	**/**

Legend: * *p* < 0.05; ** *p* < 0.01.

## Data Availability

The data presented in this study are available upon request from the corresponding author due to privacy reasons.

## Supplementary Materials

The following supporting information can be downloaded at https://www.mdpi.com/article/10.3390/children12091163/s1, Table S1: Final FAAP-O item set approbation; S2: Scoring calculator; Table S2: EFA FAAP-O.

## Abbreviations

The following abbreviations are used in this manuscript:

AIEOP	Italian Association of Paediatric Hematology and Oncology
CNS	Central Nervous System
CVR	Content Validity Ratio
EFA	Exploratory factor analysis
EP	Expert panel
FAAP-O	Functional Abilities Assessment in Pediatric Oncology
GMFM-88	Gross Motor Function Measure-88
KMO	Kaiser–Meyer–Olkin statistic
ICC	Intraclass correlation coefficients
ICF-CY	Health version for Children and Youth
TNPEEs	Neuro and psychomotor therapists of developmental age

## Appendix A. FAAP-O Final Item Set

**Table A1:** FAAP-O scale—scoresheet.

Gmfm-88 Item	FAAP-O ITEM NUMBER	Starting Position	Item Description	Scoring				
						
	**Dimension 1—supine position**			**0**	**1**	**2**	**3**	**NT**
***4***	***1***	Supine	Flexes right hip and knee through full range					
***5***	***2***	Supine	Flexes left hip and knee through full range					
***6***	***3***	Supine	Reaches out with right arm, hand crosses midline toward toy					
***7***	***4***	Supine	Reaches out with left arm, hand crosses midline toward toy					
	**Dimension 2—rolling and achievement of sitting position**			**0**	**1**	**2**	**3**	**NT**
***8***	***5***	Supine	Rolls to prone over right side					
***9***	***6***	Supine	Rolls to prone over left side					
***14***	***7***	Prone	Rolls to supine over right side					
***15***	***8***	Prone	Rolls to supine over left side					
***19***	***9***	Supine	Rolls to right side, attains sitting					
***20***	***10***	Supine	Rolls to left side, attains sitting					
	**Dimesion 3—sitting and standing position: achieved and maintenance**			**0**	**1**	**2**	**3**	**NT**
***25***	***11***	Sit on mat with small toy in front	Leans forward, touches toy, re-erects without arm propping					
***34***	***12***	Sit on bench	Maintains the position, arms and feet free, 10 s					
***59***	***13***	Sit on small bench	Attains standing without using arms					
***53***	***14***	Standing	Maintains, arms free, 3 s					
***35***	***15***	Standing	Attains sitting on a small bench					
	**Dimesion 4—moving on mat**			**0**	**1**	**2**	**3**	**NT**
***62***	***16***	Standing	Lowers to sitting on floor with control, arms free					
***42***	***17***	4 point	Reaches forward with right arm, hand above shoulder level					
***43***	***18***	4 point	Reaches forward with left arm, hand above shoulder level					
***45***	***19***	4 point	Crawls reciprocally forward 1.8m (6ft)					
***48***	***20***	Sit on mat	Attains high kneeling using arms, maintains, arms free, 10 s					
***49***	***21***	High kneeling	Attains half kneeling on left knee using arms, maintains, arms free, 10 s					
***50***	***22***	High kneeling	Attains half kneeling on right knee using arms, maintains, arms free, 10 s					
***60***	***23***	High kneeling	Attains standing through half kneeling on right knee, without using arms					
***61***	***24***	High kneeling	Attains standing through half kneeling on left knee, without using arms					
***36***	***25***	On the floor	Attains sitting on small bench					
	**Dimension 5—complex skills and movements in standing position**			**0**	**1**	**2**	**3**	**NT**
***57***	***26***	Standing	Lifts left foot, arms free, 10 s					
***58***	***27***	Standing	Lifts right foot, arms free, 10 s					
***68***	***28***	Standing, 1 hand held	Walks forward 10 steps					
***70***	***29***	Standing	Walks forward 10 steps, stops, turns 180°, returns					
***74***	***30***	Standing	Walks forward 10 consecutive steps on a straight line 2cm (3/4") wide					
***77***	***31***	Standing	Runs 4.5m (15’), stops and returns					
***81***	***32***	Standing	Jumps forward 30 cm (12"), both feet simultaneously					
***84***	***33***	Standing, holding 1 rail	Walks up 4 steps, holding 1 rail, alternating feet					
***85***	***34***	Standing, holding 1 rail	Walks down 4 steps, holding 1 rail, alternating feet					
***86***	***35***	Standing	Walks up 4 steps, arms free, alternating feet					
***87***	***36***	Standing	Walks down 4 steps, arms free, alternating feet					
	General scoring categories:							
	0 = cannot maintain the starting position or cannot perform the task (due to medical prescription or to lines or to other devices restriction) or does not initiate the task being tested. 1 = initiates but completes less than 10% of the task or completes the task as outlined in the criterion description. 2 = partially completes (10% to less than 100%) of the task or complete the task as outlined in the criterion descriptions 3 = completes the task as outlined in the criterion descriptions NT = not tested The scoring key is meant to be a general guideline. However, most of the items have specific descriptor for each score. Is it imperative that the guidelines contained in the GMFM-88 manual be used for scoring each item. The manual is sold through McKeith Press and has the information necessary for accurate and reliable scoring of the items. Items of Dimensions 1 and 2 can be performed on the bed, such as items 11 and 12 of Dimension 3, and item 17-22 of Dimension 4.							
	Scoring norms:							
	Not tested item: [(medium score obtained in the non-tested item’s dimension − 1)] × 50 Dimension 1—supine position: [(Total dimension 1) ÷ 12] × 100 Dimension 2—rolling and achievement of sitting position: [(Total dimension 2) ÷ 18] × 100 Dimension 3—sitting and standing position: achieved and maintenance: [(Total dimension 3) ÷ 15] × 100 Dimension 4—moving on mat: [(Total dimension 4) ÷ 30] × 100 Dimension 5—complex skills and movements in standing position: [(Total dimension 5) ÷ 33] × 100 Total score: [(%dimension 1 + %dimension 2 + %dimension 3 + % dimension 4 + % dimension 5) ÷ 5] × 100							
	General administration instructions:							
	If the child performs the task in a way that does not fit any of the score categories descriptions, you must choose the lowest score that best describes the observed performance. Each item could be repeated 3 times to allow the child to achieve the maximum score.							
