# Supplementary material for: Development of Functional Abilities Assessment in Paediatric Oncology (FAAP-O) Scale for Children and Adolescents Affected by Cancer
Source: Children (Basel). 2025 Sep 1;12(9):1163. doi: 10.3390/children12091163 (PMC12468092; doi:10.3390/children12091163)
Supplement: Supplementary file 1 [file children-12-01163-s001.zip › Supplementary.pdf]

Supplemental Table 1: Final FAAP-O item set approbation.

	STARTING POSITION	*ITEM*	% Confirmative votation
		**DIMENSION A**	
1.	SUPINE, HEAD IN MIDLINE	Turns head with extremities symmetrical	**100%**
2.	SUPINE	brings hands to midline, fingers one with the other	**100%**
3.	SUPINE	Lifts head 45°	**92,9%**
4.	SUPINE	Flexes right hip and knee through full range	**100%**
5.	SUPINE	Flexes left hip and knee through full range	**100%**
6.	SUPINE	Reaches out with right arm, hand crosses midline toward toy	**92,9%**
7.	SUPINE	Reaches out with left arm, hand crosses midline toward toy	**92,9%**
8.	SUPINE	Rolls to prone over right side	**92,9%**
9.	SUPINE	Rolls to prone over left side	**92,9%**
10.	PRONE	Lifts head upright	**100%**
11.	PRONE ON FOREARMS	Lifts head upright, elbows extended, chest raised	**100%**
12.	PRONE ON FOREARMS	Weight on right forearm, fully extends opposite arm forward	**92,9%**
13.	PRONE ON FOREARMS	Weight on left forearm, fully extends opposite arm forward	**92,9%**
14.	PRONE	Rolls to supine over right side	**85,7%**
15.	PRONE	Rolls to supine over left side	**85,7%**
16.	PRONE	Pivots to right 90° using extremities	**100%**
17.	PRONE	Pivots to left 90° using extremities	**100%**
		**DIMENSION B**	
18.	SUPINE, HANDS GRASPED BY EXAMINER	Pulls self to sitting with head control	**100%**
19.	SUPINE	Rolls to right side, attains sitting	**100%**
20.	SUPINE	Rolls to left side, attains sitting	**100%**
21.	SIT ON MAT, SUPPORTED AT THORAX BY THERAPIST	Lifts head upright, maintains 3 seconds	**100%**
22.	SIT ON MAT, SUPPORTED AT THORAX BY THERAPIST	Lifts head to midline, maintains 10 seconds	**100%**
23.	SIT ON MAT, ARM(S) PROPPING	Maintains 5 seconds	**100%**
24.	SIT ON MAT	Maintains, arms free, 3 seconds	**85,7%**
25.	SIT ON MAT WITH SMALL TOY IN FRONT	Leans forward, touches toy, re-erects without arm propping	**78,6%**
26.	SIT ON MAT	Touches toy placed 45° behind child's right side, returns to start	**100%**
27.	SIT ON MAT	Touches toy placed 45° behind child's left side, returns to start	**100%**
28.	RIGHT SIDE SIT	Maintains, arms free, 5 seconds	**85,7%**
29.	LEFT SIDE SIT	Maintains, arms free, 5 seconds	**85,7%**
30.	SIT ON MAT	Lowers to prone with control	**100%**
31.	SIT ON MAT WITH FEET IN FRONT	Attains 4 point over right side	**92,9%**
32.	SIT ON MAT WITH FEET IN FRONT	Attains 4 point over left side	**92,9%**

33.	SIT ON MAT	Pivots 90°, without arms assisting	**100%**
34.	SIT ON BENCH	Maintains the position, arms and feet free, 10 seconds	**100%**
35.	STANDING	Attains sitting on a small bench	**92,9%**
36.	ON THE FLOOR	Attains sitting on small bench	**78,6%**
37.	ON THE FLOOR	Attains sitting on large bench	100%
		**DIMENSION C**	
38.	PRONE	Creeps forward 1.8m (6ft)	**100%**
39.	4 POINT	Maintains weight on hands and knees, 10 seconds	**100%**
40.	4 POINT	Attains sitting arms free	**92,9%**
41.	PRONE	Attains 4 point, weight on hands and knees	**100%**
42.	4 POINT	Reaches forward with right arm, hand above shoulder level	**78,6%**
43.	4 POINT	Reaches forward with left arm, hand above shoulder level	**78,6%**
44.	4 POINT	Crawls or hitches forward 1.8m (6ft)	92,9%
45.	4 POINT	Crawls reciprocally forward1.8m (6ft)	**78,6%**
46.	4 POINT	Crawls up 4 steps on hands and knees/feet	**100%**
47.	4 POINT	Crawls backwards down 4 steps on hands and knees/feet	**100%**
48.	SIT ON MAT	Attains high kneeling using arms, maintains, arms free, 10 seconds	**100%**
49.	HIGH KNEELING	Attains half kneeling on right knee using arms, maintains, arms free, 10 seconds	**92,9%**
50.	HIGH KNEELING	Attains half kneeling on left knee using arms, maintains, arms free, 10 seconds	**92,9%**
51.	HIGH KNEELING	Kneel walks forward 10 steps, arms free	**92,9%**
		**DIMENSION D**	
52.	ON THE FLOOR	Pulls to stand at large bench	**85,7%**
53.	STANDING	Maintains, arms free, 3 seconds	**100%**
54.	STANDING	Holding onto large bench with one hand, lifts right foot, 3 second	**100%**
55.	STANDING	Holding onto large bench with one hand, lifts left foot, 3 seconds	**100%**
56.	STANDING	Maintains, arms free, 20 seconds	**71,4%**
57.	STANDING	Lifts left foot, arms free, 10 seconds	**100%**
58.	STANDING	Lifts right foot, arms free, 10 seconds	**100%**
59.	SIT ON SMALL BENCH	Attains standing without using arms	**100%**
60.	HIGH KNEELING	Attains standing through half kneeling on right knee, without using arms	**100%**
61.	HIGH KNEELING	Attains standing through half kneeling on left knee, without using arms	**100%**
62.	STANDING	Lowers to sitting on floor with control, arms free	**100%**
63.	STANDING	Attains squat, arms free	**92,9%**
64.	STANDING	Picks up object from floor, arms free, returns to standing	**92,9%**
		**DIMENSION E**	
65.	STANDING 2 HANDS ON LARGE BENCH	Cruises 5 steps to right	**92,9%**
66.	STANDING 2 HANDS ON LARGE BENCH	Cruises 5 steps to left	**92,9%**

67.	STANDING 1 HAND HELD	Walks forward 10 steps	**100%**
68.	STANDING 1 HAND HELD	Walks forward 10 steps	**100%**
69.	STANDING	Walks forward 10 steps	**100%**
70.	STANDING	Walks forward 10 steps, turns 180°, returns	**100%**
71.	STANDING	Walks backward10 steps	**100%**
72.	STANDING	Walks forward 10 steps, carrying a large object with 2 hands	**92,9%**
73.	STANDING	Walks forward 10 consecutive steps between parallel lines 20cm (8") apart	**78,6%**
74.	STANDING	Walks forward 10 consecutive steps on straight line 2cm (3/4") wide	**100%**
75.	STANDING	Steps over stick at knee level, right foot leading	**92,9%**
76.	STANDING	Steps over stick at knee level, left foot leading	**92,9%**
77.	STANDING	Runs 4.5m (15'), stops and returns	**78,6%**
78.	STANDING	Kicks ball with right foot	**85,7%**
79.	STANDING	Kicks ball with left foot	**85,7%**
80.	STANDING	Jumps 30cm (12in) high, both feet simultaneously	**78,6%**
81.	STANDING	Jumps forward 30cm (12in), both feet simultaneously	**92,9%**
82.	STANDING	Hops on right foot 10 times within a 60 cm (24in) circle	**92,9%**
83.	STANDING	Hops on left foot 10 times within a 60 cm (24in) circle	**100%**
84.	STANDING, HOLDING 1 RAIL	Walks up 4 steps, holding 1 rail, alternating feet	**100%**
85.	STANDING, HOLDING 1 RAIL	Walks down 4 steps, holding 1 rail, alternating feet	**100%**
86.	STANDING	Walks up 4 steps, arms free, alternating feet	**100%**
87.	STANDING	Walks down 4 steps, arms free, alternating feet	**100%**
88.	STANDING ON 15-18CM (6-7IN) STEP	Jumps off, both feet simultaneously	**100%**

Supplementary table 2 – EFA FAAP-O

**ITEM GMFM-88**	**STARTING POSITION**	**ITEM DESCRIPTION**	**EFA Values for each DIMENSION**				
			E	D	C	B	A
4.	SUPINE	Flexes right hip and knee through full range	,143	,186	,117	,133	**,815**
5.	SUPINE	Flexes left hip and knee through full range	,150	,130	,206	,202	**,840**
6.	SUPINE	Reaches out with right arm, hand crosses midline toward toy	,088	,015	,081	,222	**,875**
7.	SUPINE	Reaches out with left arm, hand crosses midline toward toy	,109	,041	,135	,296	**,807**
8.	SUPINE	Rolls to prone over right side	,204	,261	,188	**,737**	,370
9.	SUPINE	Rolls to prone over left side	,243	,306	,225	**,701**	,335

14.	PRONE	Rolls to supine over right side	,252	,367	,162	**,737**	,302
15.	PRONE	Rolls to supine over left side	,261	,352	,201	**,727**	,291
19.	SUPINE	Rolls to right side, attains sitting	,243	,166	,399	**,663**	,254
20.	SUPINE	Rolls to left side, attains sitting	,190	,197	,413	**,685**	,167
25.	SIT ON MAT WITH SMALL TOY IN FRONT	Leans forward, touches toy, re-erects without arm propping	,074	,233	**,579**	,286	,292
34.	SIT ON BENCH	Maintains the position, arms and feet free, 10 seconds	,078	,218	**,582**	,324	,425
35.	STANDING	Attains sitting on a small bench	,274	,403	**,682**	,252	,193
36.	ON THE FLOOR	Attains sitting on small bench	,376	**,585**	**,426**	,170	,120
42.	4 POINT	Reaches forward with right arm, hand above shoulder level	,188	**,703**	,304	**,416**	,125
43.	4 POINT	Reaches forward with left arm, hand above shoulder level	,191	**,716**	,327	,395	,146
45.	4 POINT	Crawls reciprocally forward 1.8m (6ft)	,228	**,717**	,226	,224	-,035
48.	SIT ON MAT	Attains high kneeling using arms, maintains, arms free, 10 seconds	,247	**,773**	,348	,188	,180
49.	HIGH KNEELING	Attains half kneeling on right knee using arms, maintains, arms free, 10 seconds	,467	**,675**	,223	,261	,199
50.	HIGH KNEELING	Attains half kneeling on left knee using arms, maintains, arms free, 10 seconds	,477	**,674**	,229	,244	,199
53.	STANDING	Maintains, arms free, 3 seconds	,200	,387	**,758**	,242	,014
57.	STANDING	Lifts left foot, arms free, 10 seconds	**,677**	,077	,496	,184	,108
58.	STANDING	Lifts right foot, arms free, 10 seconds	**,660**	,114	,467	,228	,105
59.	SIT ON SMALL BENCH	Attains standing without using arms	,405	,391	**,591**	,325	,144
60.	HIGH KNEELING	Attains standing through half kneeling on right knee, without using arms	**,520**	**,657**	,218	,205	,067
61.	HIGH KNEELING	Attains standing through half kneeling on left knee, without using arms	**,504**	**,669**	,240	,131	,106
62.	STANDING	Lowers to sitting on floor with control, arms free	**,519**	**,579**	,370	,143	,018
68.	STANDING 1 HAND HELD	Walks forward 10 steps	**,479**	,361	**,745**	,170	,189

70.	STANDING	Walks forward 10 steps, turns 180°, returns	**,438**	,388	**,625**	,173	,126
74.	STANDING	Walks forward 10 consecutive steps on straight line 2cm (3/4in) wide	**,753**	,100	,258	,196	,099
77.	STANDING	Runs 4.5m (15ft), stops and returns	**,680**	,331	,022	,155	,129
81.	STANDING	Jumps forward 30cm (12in), both feet simultaneously	**,662**	,395	,076	,144	,122
84.	STANDING, HOLDING 1 RAIL	Walks up 4 steps, holding 1 rail, alternating feet	**,711**	,373	,228	,174	,075
85.	STANDING, HOLDING 1 RAIL	Walks down 4 steps, holding 1 rail, alternating feet	**,761**	,347	,220	,153	,145
86.	STANDING	Walks up 4 steps, arms free, alternating feet	**,851**	,240	,065	,123	,103
87.	STANDING	Walks down 4 steps, arms free, alternating feet	**,882**	,178	,098	,112	,099

Legend:

- black values → EFA > 0,40
- grey values → EFA values near to 0,40, but still lower
